# Left Ventricle Diverticulum with Partial Cantrell's Syndrome

**DOI:** 10.1155/2012/309576

**Published:** 2012-11-26

**Authors:** Mustapha El Kouache, S. Labib, A. El Madi, A. Babakhoya, S. Atmani, Y. Abouabdilah, M. Harandou

**Affiliations:** ^1^Unit of Cardiac Pediatric Surgery, Department of Pediatric Surgery, Faculty of Medicine, Hassan II University Hospital, 30000 Fez, Morocco; ^2^Department of Anatomy, Faculty of Medicine, Hassan II University Hospital, 30000 Fez, Morocco; ^3^Department of Anesthesia and Reanimation, Hassan II University Hospital, 30000 Fez, Morocco; ^4^Cardiac Pediatric Department, Hassan II University Hospital, 30000 Fez, Morocco

## Abstract

Cantrell syndrome is a very rare congenital disease associating five features: a midline, upper abdominal wall disorder, lower sternal abnormality, anterior diaphragmatic defect, diaphragmatic pericardial abnormality, and congenital abnormalities of the heart. In this paper, we report a case of partial Cantrell's syndrome with left ventricular diverticulum, triatrial situs solitus, ventricular septal defect, dextrorotation of the heart, an anterior pericardial diaphragmatic defect, and a midline supraumbilical abdominal wall defect with umbilical hernia. The 5-month-old patient underwent a successful cardiac surgical procedure. A PTFE membrane was placed on the apex of the heart to facilitate reopening of the patient's chest. Postoperative course was uneventful. The patient was discharged with good clinical condition and with a normal cardiac function.

## 1. Introduction

Cantrell syndrome is a very rare congenital disease associating five features: a midline, upper abdominal wall disorder, lower sternal abnormality, anterior diaphragmatic defect, diaphragmatic pericardial abnormality, and congenital abnormalities of the heart. We present this rare case to show the diagnostic and therapeutic particularities of this congenital disease.

## 2. Case Report

**Figure 1:**
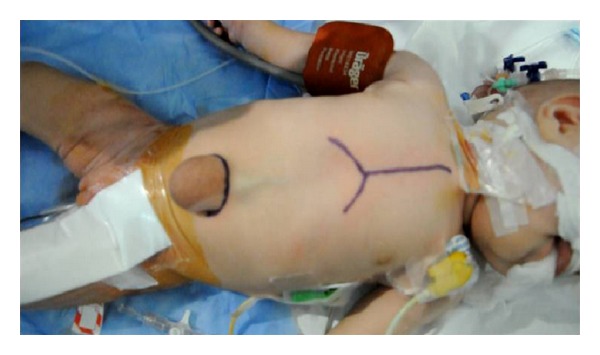
The patient while prepared for surgery. Pulsatile supraumbilical mass which was initially diagnosed as umbilical hernia.

**Figure 2:**
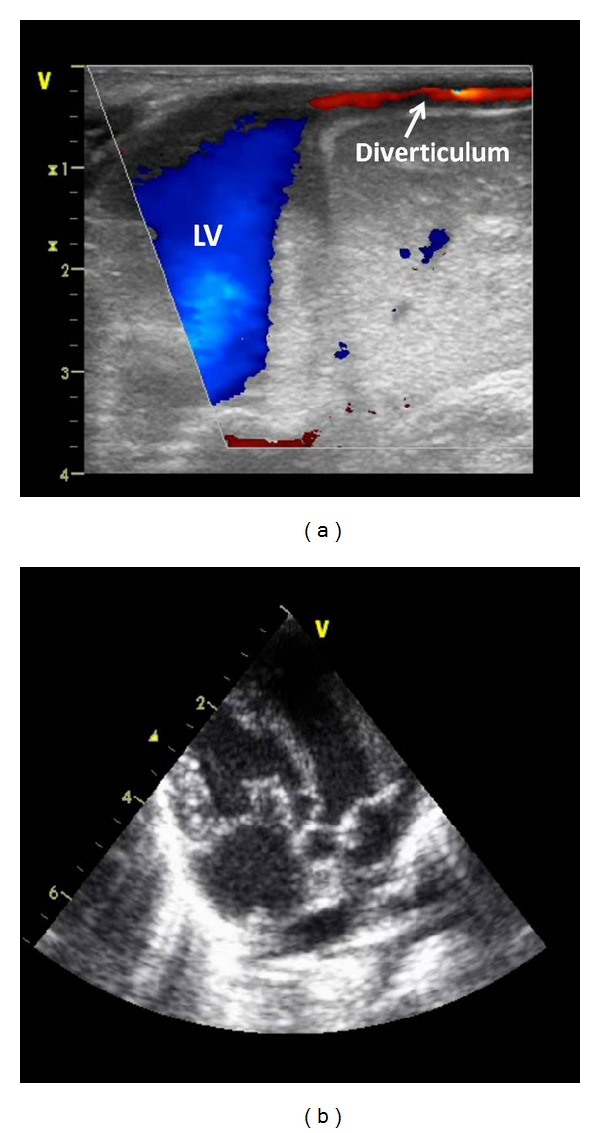
The echocardiography demonstrated blood flow in and out of the pulsatile mass, a left ventricle diverticulum, dextrorotation ofthe heart, a triatrial situs solitus, a small ventricular septal defect, and normal venous anatomy were demonstrated.

A 3-month-old female has presented with a prominent pulsatile supraumbilical mass which was initially diagnosed as umbilical hernia. The patient was the first birth of the mother after 39 gestation weeks and weighing 2.800 kg. The delivery was normal and without noticeable event. The physical examination showed a pulsatile mass with a thoracoabdominal wall defect, the xyphoid was absent, and the sternum was short (Figure 1). The heart rate and rhythm were normal with 100% of oxygen saturation. The clinical examination showed a subcutaneous pulse-synchronous pulsatile mass with umbilical hernia. The echocardiography (Figure 2) and helical computed tomography (CT) demonstrated blood flow in and out of the pulsatile mass. In addition, a left ventricule diverticulum, dextrorotation of the heart, a tri-atrial situs solitus, a small ventricular septal defect, and normal venous anatomy were demonstrated. The atrial septal deficiency and patent ductus arteriosus were shown.

**Figure 3:**
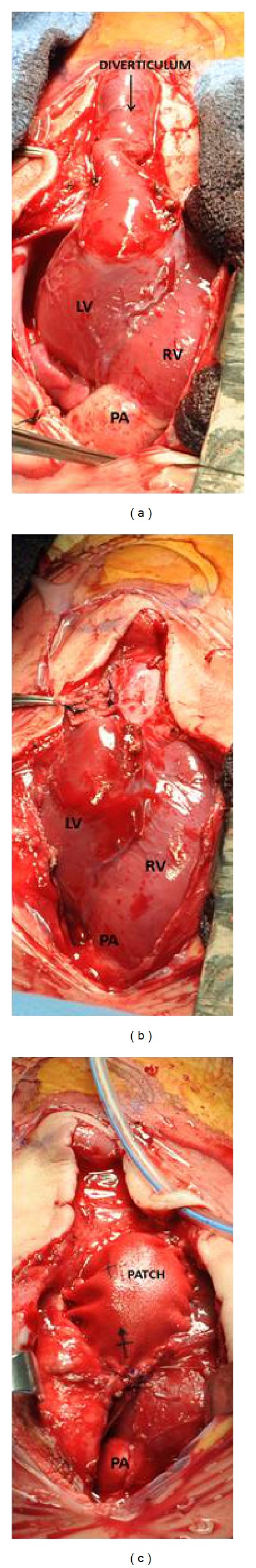
Thoracoabdominal incision; contracting heart while all components are shown. LV: left ventricle, RV: right ventricle, PA:pulmonary artery.

The chromosome study showed a normal female karyotype. The infant underwent surgery while being 5 months old with a general anesthetic. Surgical resection of the LV diverticulum was performed without the use of cardiopulmonary bypass through a sternotomy and a midline incision; exposure from the apex of the heart along the length of the mass from the sternum to the umbilicus was achieved. Once the diverticulum was fully exposed, a series of pledgeted sutures were used to ligate the diverticulum at the apex of the heart. The pulsatile movement of the mass then ceased. The diverticulum was excised. Overlapping reconstruction of the abdominal wall without the use of any prosthetic material was performed. A PTFE membrane was placed over the apex of the heart to facilitate reopening of the patient's chest (Figure 3). Postoperative course was uneventful. The patient was discharged in good clinical condition and with a normal cardiac function.

## 3. Discussion

Cantrell syndrome, alternatively known as pentalogy of Cantrell, is a rare congenital disease with multiple disorders associated with midline development [1]. The full expression of the Cantrell pentalogy or thoracoabdominal ectopia cordis includes a ventricular septal disorder, left ventricular diverticulum, dextrocardia, and an abdominal wall disorder mostly expressed in the form of omphalocele, and less frequently gastroschisis [2]. Usually, there is an adjacent anterior diaphragmatic hernia is found. The clinical presentation is ranging from soft forms similar to our case to severe disorder involving omphalocele, ectopia cordis. The most severe congenital heart disorder involves tetralogy of Fallot or hypoplastic left heart syndrome (HPLHS) [3]. The diagnosis of pentalogy of Cantrell is achieved by the antenatal ultrasound in the pregnancy first trimester. The accurate early anatomic assessment of components of this syndrome is crucial for optimal parental orientation and decision making on the outcome of pregnancy. The antenatal and cardiac ultrasound and magnetic resonance imaging whenever necessary have to be used for divulging combination of malformations. It is important to distinguish the congenital left ventricular diverticulum from an aneurysm. The wall of true congenital diverticulum is formed of three cardiac layers and contracts normally whereas an aneurysm is generally a fibrous saccular lesion with paradoxical contraction [4]. This patient has had an incomplete expression of pentalogy of Cantrell because of the absence of both a diaphragmatic hernia. The surgical intervention consisted of corrective or palliative cardiovascular surgery, correction of ventral hernia and diaphragmatic disorder, and correction of associated abnormalities. Generally, the pentalogy of Cantrell is a fatal without surgery. Our patient underwent successful surgery despite its high mortality rate. 

It is commonly recommended that initial surgery treats thoracoabdominal disorder when these are not restrictive, and that heart lesions be corrected later [5]. Nevertheless, the surgical intervention is the treatment of choice to prevent potential complications. The best treatment strategy depends on the size of the abdominal wall disorder, the associated heart anomalies, and the type of ectopia cordis [6].

## 4. Conclusion

Left ventricle diverticulum with partial Cantrell's syndrome is a very rare congenital disease. Diagnosis is relatively easy; the treatment is always surgical with good postoperative prognosis.
